# A quantitative methodology for measuring the social sustainability of pavement deterioration

**DOI:** 10.1038/s41598-024-52655-7

**Published:** 2024-01-24

**Authors:** Egemen Okte, Jessica Boakye, Mark Behrend

**Affiliations:** 1 Civil and Environmental Engineering, University of Massachusetts Amherst, 130 Natural Resources Rd, Amherst, MA 01003 USA; 2 Mechanical Engineering, Rochester Institute of Technology, 1 Lomb Memorial Dr, Rochester, NY 14623 USA

**Keywords:** Civil engineering, Sustainability

## Abstract

The social pillar of pavement sustainability is understudied compared to economic and environmental pillars, making it difficult to integrate into life-cycle methodologies. While methods such as social life cycle assessment (S-LCA) exist, they usually focus on social governance rather than quantifying the impact of pavement investment decisions on communities. This study introduces a methodology to quantify the impact of road condition on vulnerable communities, specifically Environmental Justice (EJ) communities. The methodology calculates the impact of road condition on residents and analyzes fuel consumption (as an example impact) for road users during recurrent home-work trips as a function of pavement condition for EJ and non-EJ communities. It was found that EJ communities in Massachusetts are twice as likely to live near poor condition roads and consume twice as much excessive fuel during recurrent home-work trips. The proposed method is designed to integrate into existing life-cycle methods and represents a significant step towards integrating equity into pavement management decisions.

## Introduction

Transportation is the largest sector for greenhouse gas (GHG) emissions in the US with about 29%^1^. Given that freight transportation is responsible for nearly 70% of the total domestic freight volume^2^ and personal vehicles make up 87% of passenger transportation by mode^3^, pavements are one of the most integral parts of transportation sustainability as pavements are necessary to support the movement of people and goods in modern society. Pavement sustainability can be defined as the pavement’s ability to: achieve engineering goals; preserve and restore surrounding ecosystems; use resources economically; and meet basic human needs such as health, safety, equity, employment, comfort, and happiness^4^. This has also been referred to as the triple-bottom line or the three pillars of sustainability which focus on economic, environmental, and social sustainability^5^. The economic and environmental definitions have been well studied in the literature and can be quantified through life-cycle cost analysis (LCCA) and life-cycle assessment (LCA) respectively.

Life cycle cost analysis (LCCA) is often used to assess economic sustainability incurred by agency (e.g., construction, maintenance, and rehabilitation) and user (e.g., vehicle operation, wear-tear, crash, delay) costs throughout the pavement’s lifetime^6,7^. These costs are calculated by considering both the present and anticipated future expenses using deterministic or probabilistic methods^6,8^. In determining the present value of future costs, all expenses are adjusted to the present year using a discount rate. The discount rate used may be updated according to the rate provided by the Office of Management and Budget, a Federal Government agency, although each agency or stakeholder is incentivized to select their own discount rate depending on the analysis^9,10^. As LCCA is a comparative tool by nature, it is often used to evaluate different pavement alternatives and optimize to choose the most cost-effective option^11–14^.

Life cycle assessment (LCA) is used to assess the potential environmental impacts and resources consumed throughout a pavement’s lifetime. Pavement life-cycle is usually broken down to materials, construction, maintenance, use, and the end-of-life stages^15–18^ and an LCA analysis may cover one or many of these stages. LCA is generally made up of four steps which are goal and scope, inventory analysis, impact assessment, and interpretation^16^. One of the widely recognized and used methods in environmental impact assessment is the Tool for the Reduction and Assessment of Chemical and other environmental Impacts (TRACI) developed by EPA to quantify various environmental impacts such as climate change, ozone depletion and others^19^. LCA provides a framework for evaluating and comparing the environmental impacts of different systems, including transportation infrastructure. The use of LCA for pavement design and maintenance is also well documented in the literature as it can be used to measure the impact of different materials, agency decisions and pavement alternatives^13,20–25^. One of the most recent uses of LCA is through environmental product declarations (EPD’s) which provides basic environmental impact information related to the production of a specific material production^17,26,27^ often compared to food labels on packaged food products.

The majority of pavement sustainability studies focused on environmental and economic components of sustainability, especially after the Federal Highway Administration (FHWA) launched the Sustainable Pavements Program in 2010 to better understand and promote pavement sustainability in practice. Through this program, technical briefs and tools have been introduced which document life-cycle methods that state agencies and local governments can use to assess and improve pavement sustainability (with tools such as RealCost for LCCA and LCAPave for LCA)^28–30^.

The most understudied aspect of pavement sustainability is the social dimension. The assessment of social sustainability requires the development of metrics which can quantify the impact of pavement design, rehabilitation, and maintenance decisions on communities and people of interest. Special attention should be given to groups which may disproportionately face negative impacts. For instance, the California Office of Environmental Health Hazard Assessment found that minority and low-income residents were more likely to live near congested highways, which has been shown to lead to adverse health effects. As a result, traffic impact is one of the indicators regularly measured by California and used to inform community investment decisions through the CalEnviroScreen Tool^31^. However, such indicators are not directly related to pavement management decisions such as deterioration due to deferred maintenance, which can further increase emissions and fuel consumption^32,33^. S-LCA for pavements has been also proposed as a tool to help quantify the social dimension of sustainability as it relates to pavements and infrastructure, similar to economic and environmental dimensions of sustainability. However, S-LCA generally focuses on more on social governance and human well-being throughout the structure’s life-cycle rather than quantitatively assessing the impact of pavement investment decisions on individuals. For example, studies have used scoring systems and defined social indicators such as management style, stakeholder engagement, and worker safety for pavement projects^34,35^. More recently, quantitative methods have been proposed to better understand equity impacts around pavement decisions. Recent work has found that pavement is likely to be in poor condition near census tracts with racial and ethnic minorities, low-income, and urban areas^36^ and the city of Oakland distributes local funding for local streets by the share of under-served populations and share of local street miles in poor condition^37^. Other research has proposed optimization techniques to try to minimize the number of poor conditioned roads in disadvantaged communities^38–40^. The importance of transportation services and the consequences of inaccessibility have been recently studied in hazard and risk/resilience analysis contexts^41,42^. This study aims to build upon recent work in pavement sustainability and transportation equity by (1) confirming and expanding on the finding/significance that disadvantaged communities have disproportionate amounts of poor roads, (2) calculating the disproportional impact of deteriorated roads during essential recurring trips (i.e., home-work) and, (3) discussing how this method could be used to improve holistic pavement sustainability assessment, including the use of LCA and LCCA.

## Goal and scope of the paper

**Figure 1:**
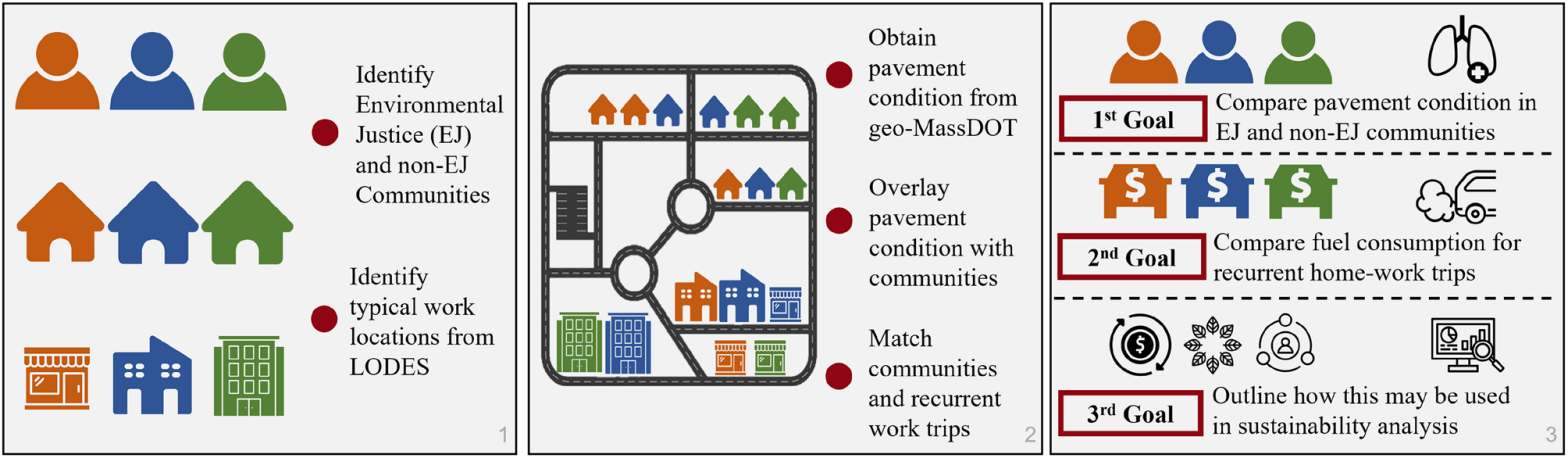
Outline of this study.

This paper introduces a methodology that quantitatively describes and measures the impact of road condition on vulnerable communities and shows the importance of considering the social component in pavement sustainability assessment. The state of Massachusetts is used as an example case area. There are three main goals, which are shown in Fig. 1 and also listed below:

1. Calculating the amount of poor roads inside and outside disadvantaged communities and discussing the impacts of living near deteriorated roads.
2. Calculating the amount of extra fuel (energy) consumption due to deteriorated roads during essential and recurring home-work trips originating inside and outside disadvantages communities.
3. Describing how this methodology could be used to improve pavement sustainability, including incorporating social impacts into LCA and LCCA frameworks.

Within this study, disadvantaged communities are noted as Environmental Justice (EJ) communities. This term is used in this work for two main reasons. First, to recognize that disproportionate pavement condition and its subsequent impact is part of a larger problem and historical trend in the United States of unequal impacts of environmental pollution on different social classes and racial/ethnic groups^43^. Second, the state of Massachusetts, through the Massachusetts Department of Transportation, defines EJ communities and provides the necessary geographic information^44^. Past work has focused only on roads within vulnerable populations but has not studied the roads that vulnerable populations need for essential trips. This consideration is important, as focusing only on poor roads in vulnerable communities may not provide a complete picture on the disproportionate impact. Therefore, work-home trips are proposed to identify additional roads that are important for EJ communities. These trips are chosen because work-home trips are frequent and therefore, the impact of road condition on these trips are more important compared to less frequent trips. Pavement conditions within and used by EJ communities are then used to calculate disproportionate poor roads and additional fuel consumption. It should be noted that while fuel consumption is not the only impact that could be focused on, it was selected to demonstrate the methodology. This method is proposed as a first step in creating a more holistic quantification of road impacts.

The rest of the manuscript is organized is follows. The next section introduces the proposed methodology and illustrates the methodology through a case study of Massachusetts, and discusses the assumptions and limitations of the method. The third section presents the results of the study including an analysis of the road condition in EJ communities, impact of road condition on home-work trips, and the use of this method for future sustainability analysis. The final section summarizes this study.

## Methodology

### Description

The methodology includes four steps: (1) Definition of study area and communities of interest, (2) Creation of a geographical network using pavement data, (3) Definition of trips of interest, and (4) Distributional analysis of societal impacts. We discuss each of these steps in detail below.

#### Step 1: Definition of study area and communities of interest

The study area should be defined based on the granularity of data available and general study goals. Within the study area, detailed pavement deterioration data must be obtained. For example, a district which has detailed deterioration data on local roads may want to restrict the study area to the district. In contrast, state agencies may want to understand the distributional inequities within their state and which would require the state to have statewide deterioration data.

Within the study area of interest, populations of interest or vulnerable populations should be defined. The definitions of these populations will be unique for each study case. The populations are linked by geography and societal characteristic. For this context, populations which have historically faced disproportionate transportation burden such as racial minorities, low income groups, and immigrant status are especially salient^45,46^. Since these definitions vary, it is suggested that the definition of vulnerable community come from relevant decision makers and/or discussions with the community of interest to ensure that the appropriate groups have been identified.

#### Step 2: Creation of a geographical network incorporating pavement deterioration data

The road system within the community of interest must then be turned into a geographical network. Previous research has used graph theory to translate the the transportation system into a graph network and facilitate the calculation of network metrics^47^. A network is defined by a set of nodes and edges which connect the nodes to each other^48^. Within the context of a generic transportation network, nodes often mark places of interest such as residential hubs, shelters, schools, or workplaces while edges follow the topology of the road network. Nodes and edges can have properties of intensest. Nodal weights can be used to capture the distribution of the population within the study area while edge weights can be used to capture travel time or travel distance along the edge. Additionally, the pavement condition can be a property of the edges.

#### Step 3: Definition of trips of interest

Following the creation of the geographic network, trips of interest must be defined. These trips will provide information about origin and destination nodes within the study area. To quantify distributional inequity, it is important that the chosen trips be essential, regular, and measured over time. Regular, essential trips provide a stable measure of user impact which is necessary for incorporation into a life cycle assessment. Moreover, it is important that the trips chosen be measured over time such that long term trends can be established and incorporated into life-cycle calculations. For these reasons, work trips are chosen for quantifying social pavement sustainability.

#### Step 4: Distributional analysis of social impacts

Past work has shown that focusing only on economic and environmental constraints can lead to inequitable outcomes in the context of pavement management^39^. Therefore, distributional analysis has been recommended. Distributional analysis requires evaluation of the impacts for separate groups. The need for distributional analysis within policy and regulatory groups is underscored by recent the Biden-Harris Executive Order which explicitly calls for distributional analysis across different community groups^49^.

Societal metrics chosen must be influenceable (i.e., the impact will change if the pavement condition distribution changes)^50^. Influenceability allows for metrics to be optimized over a life-cycle which is the goal of sustainable pavement management. Therefore, metrics such as overall cost and travel time which can be influenced by a multitude of factors including land-use, economic opportunity, and supporting infrastructure are not considered^51^. Although these are important factors, they are often outside of the scope of pavement management decision makers. This study aims to identify factors that these decision makers can examine to make more equitable decisions.

Two societal impacts of interest for pavement deterioration are identified. The first is the distribution of pavement condition across different community groups. This impact is chosen because road condition has been linked to safety (poor condition roads have been linked to more frequent crashes) and economic prosperity (poor condition roads have been linked to lower market valuations of nearby properties)^52,53^. The second impact is the distributional fuel consumption over recurrent trips. This impact is chosen because past research has shown that vehicles have to use more fuel when they travel over poor condition roads leading directly to higher user costs^6,38^. This additional fuel consumption means the car may produce more harmful particulate matter disproportionately affecting communities with more poor condition roads (the first impact).

### Application

The four step methodology was applied to roads in Massachusetts. Each of the steps are discussed in detail below.

#### Step 1: Definition of study area and communities of interest

Since the study area is Massachusetts, the paper relies on the EJ community definition provided by the Massachusetts Department of Transportation (MassDOT). Although the paper uses the MassDOT definitions, the method is flexible and can be applied to any EJ criteria. Massachusetts defines EJ communities on a block group level of granularity and uses data provided by the United States Census^44^. A block group is a statistical division of a census tract and is generally defined to contain 600-3000 people^54^. In Massachusetts, a census block group is defined as an EJ community if it satisfies at least one of the four following conditions^44^:

1. The annual median household income is 65% or less of the statewide annual median household income
2. Minorities make up 40% or more of the population
3. 25% or more of households identify as speaking English less than “very well”
4. Minorities make up 25% or more of the population and the annual median household income of the municipality in which the neighborhood is located does not exceed 150% of the statewide annual median household income.

**Figure 2:**
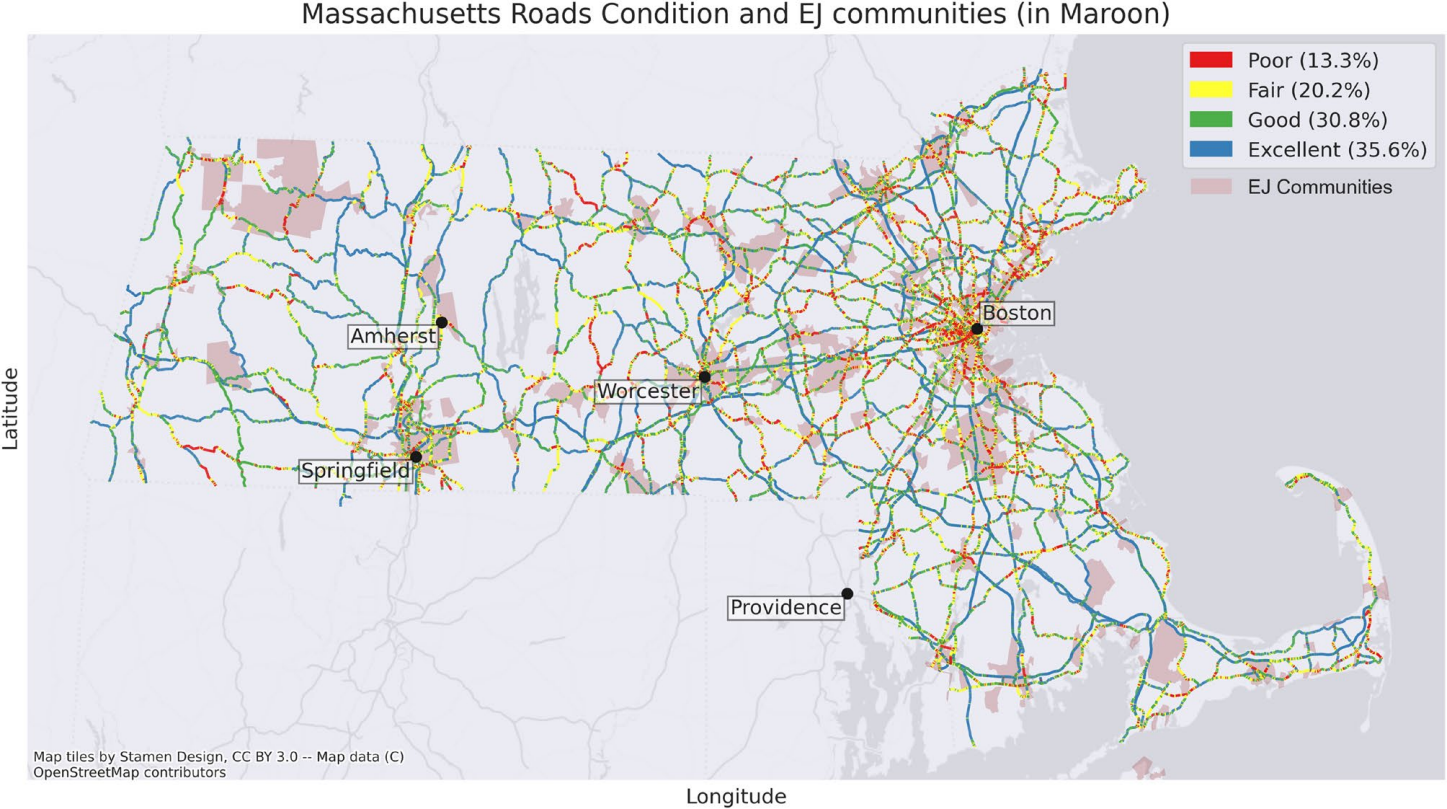
Massachusetts road condition grouped by PSI.

Figure 2 shows locations of EJ communities overlaid with roads in the state. Road data (including road type, distance, and speed) was obtained from geo-MassDOT. GeoDOT maintains and provides all GIS data that is used by MassDOT officials (demonstrating the trustworthiness of the data) and is publicly available. In Massachusetts, there are 4,985 block groups, and 2604 of these blocks have been deemed EJ communities with a total population of 3,487,681 citizens.

#### Step 2: Creation of a geographical network incorporating pavement deterioration data

In addition to the geographical locations of the roads, information on pavement condition is needed to see how varying road conditions could effect EJ and non-EJ community members in Massachusetts. The geo-MassDOT data provides condition in Present Serviceability Index (PSI) for 5967mi of the state owned roads. PSI is a measure of road quality as a mathematical function of cracking, rutting, patching and slope variance^55^. PSI ranges from 0 to 5, with a higher value indicating a higher quality road^56^. Figure 2 shows the road conditions throughout Massachusetts, together with EJ communities. Excellent, good, fair and poor roads make up approximately 36%, 31%, 20% and 13% of the roads respectively. MassDOT classifies the road condition as follows^57^:

- Poor if $$0<\text {PSI}\le 2.5$$
- Fair if $$2.5<\text {PSI}\le 3$$
- Good if $$3<\text {PSI}\le 3.5$$
- Excellent if $$3.5<\text {PSI}\le 5$$

#### Step 3: Definition of trips of interest

As one of the goals of this study is to find fuel consumption due to recurrent home-work trips, it was necessary to collect home and work locations for Massachusetts residents. The LEHD Origin-Destination (OD) Employment Statistics (LODES) from the United States Census Bureau provides data on where Americans live and work^58^. LODES provides geocodes (locations) of home and work census blocks for state residents. The subset of the LODES data used in this study only examines the origin and destination (home-work) of Massachusetts residents and the number of people taking the trips within the resolution of a census block for privacy reasons. This study only focused on OD’s for residents who live and work in Massachusetts, which are denoted in the dataset as *main* as opposed to *auxiliary* from the year 2019 (the latest year without COVID-19 impacts). The dataset contains information about 3,017,657 trips taken by 3,311,599 people. Residents with residence geocodes within EJ block groups were marked as EJ communities, and others were marked as non-EJ communities.

**Figure 3:**
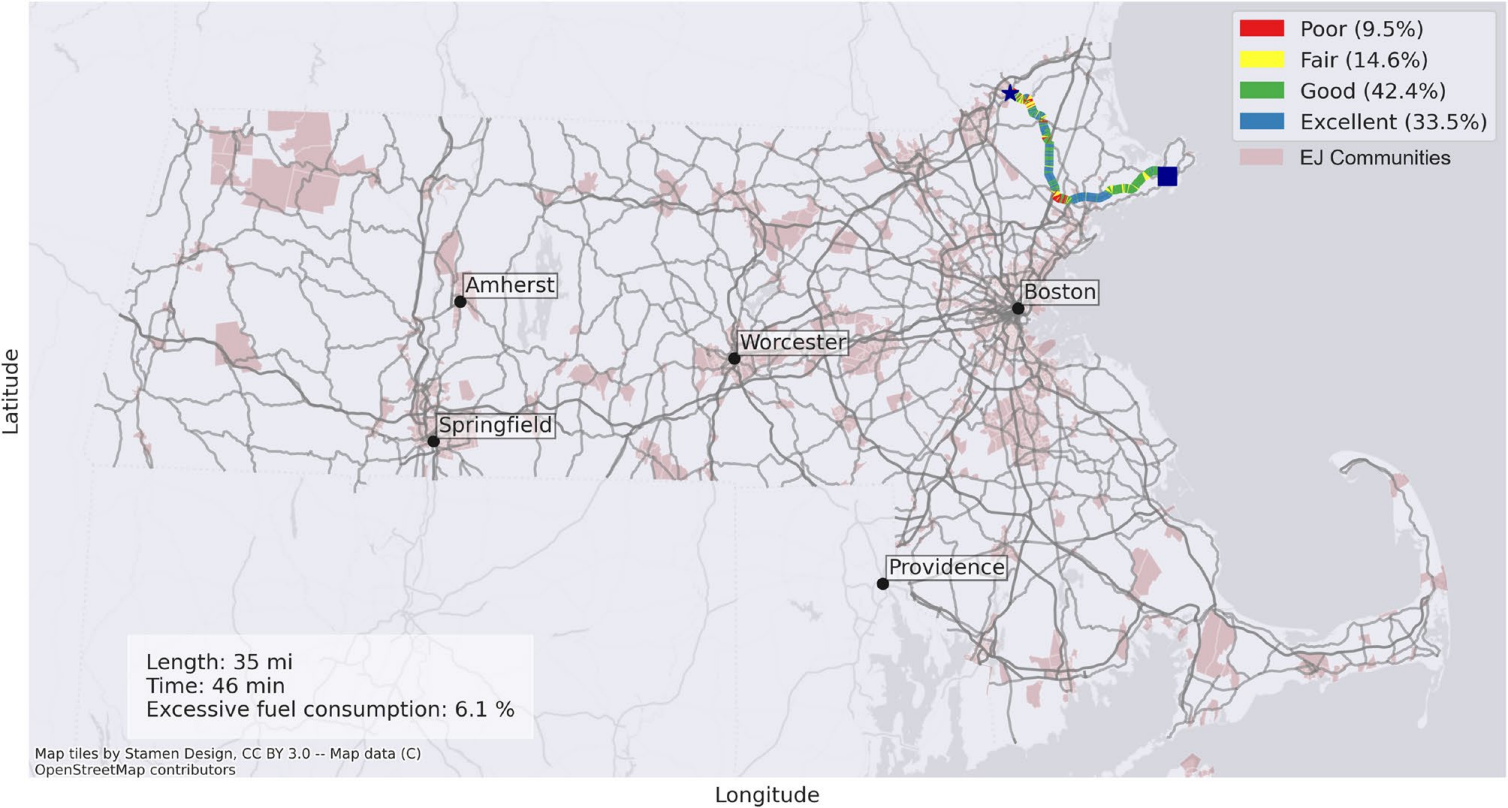
Example excessive fuel/energy calculation for an EJ start point.

Once the trips were identified, using the geo-MassDOT pavement dataset, a road graph was constructed to find the shortest path between OD points, where the shortest path is defined as the trip that takes the least amount of time. The trips under 5 miles were removed from the dataset to ensure that the dominant mode of transport was not walking and/or biking, leaving 2,985,197 trips as trips under 5 miles were under 2% of all trips. It is important to note here that the remaining trips may not have to be taken by a personal vehicle (70% of trips in Massachusetts are taken via personal vehicles while under 8% of the trips are taken via transit^59^), as the LODES dataset does not have travel mode information. However, it was assumed that it was at least possible to take these trips with personal vehicles. Finally, for all people taking the trips, their excessive fuel consumption was calculated using the excessive fuel/energy consumption in Eq. (3). Figure 3 shows a trip calculation example, with the rectangle being the home node and the star being the work node.

#### Step 4: Distributional analysis of social impacts

Two impacts are selected for analysis. The first impact is the distribution of the pavement condition within EJ and non-EJ communities. This was calculated using the EJ and road datasets obtained in step 2. The second impact is the distributional fuel consumption over recurrent trips. This was calculated as a function of pavement condition. In order to calculate fuel consumption, International Roughness Index (IRI) values were used to estimate the excessive fuel consumption. IRI is a roughness measure describing the amount of vertical movement over a unit road length. A higher IRI value indicates a higher roughness. PSI values were converted to IRI using Eq. (1), using a regression equation obtained from^60^. It is important to note that even though this conversion may introduce errors as there is no direct IRI value in the geo-MassDOT dataset, it should preserve the trends of overall fuel consumption.

1 $$\begin{aligned} IRI=\frac{50}{9} \cdot \ln \left[ \frac{5}{PSI}\right] \end{aligned}$$

The IRI value was then used to find the energy consumption of a passenger vehicle. The equation from^61^ describes this releationship using regression analysis:

2 $$\begin{aligned} E(v,IRI) = \frac{p}{v}+(k_a \cdot IRI + d_a) + b \cdot v + (k_c \cdot IRI + d_c) \cdot v^2 \end{aligned}$$

where constants $p=3.3753 \times 10^{4}$, $k_a=6.70 \times 10^{-1}$, $d_a=2.1757 \times 10^{3}$, $b=-1.6931 \times 10^{1}$, $k_c=2.81 \times 10^{-4}$, $d_c=2.1860 \times 10^{-1}$, *E* is energy (kJ/mi), *v* is average speed (mph) (speed limit in the geo-MassDOT dataset), and *IRI* is the pavement roughness (in/mi).

The final step was to calculate the excess fuel consumption due to road condition. To find the excess, energy consumption can be compared to the amount of fuel that would be used on an *ideally smooth* road. Using^6^ this ideal IRI value was assumed as 40 in/mi. Then, using equation 3, the percent excessive fuel consumption due to road conditions was calculated:

3 $$\begin{aligned} \Delta E = \frac{E(v,IRI)-E(v,40)}{E(v,40)}\cdot 100 \end{aligned}$$

It is important to note that $\Delta E$ (%) measures the percent increase in fuel consumption only due to road condition for a given route.

### Assumptions and limitations

One of the assumptions in this study was that all trips follow the MassDOT managed road network. While there are local roads outside MassDOT jurisdiction, most trips longer than 5 miles are much more likely to include major routes and interstates as part of their trip, as confirmed by Google Maps and Open Street Maps APIs. However, in the future, it may be important to break down roads by jurisdiction and only focus on the roads that the agency is responsible for. For instance, cities may be responsible for the start and end of a trip where local roads are used, and the state agency may be responsible for major roadways.

Another assumption in this study was that it is possible to take all trips longer than 5 miles by personal vehicle. Because LODES data include no information about the travel mode, it was not possible to separate personal vehicle trips with public transportation. Future work will focus on getting traveler information from MassDOT to account for travel mode choice at a much higher resolution, such as a city or county. However, it was not possible to conduct this analysis at a state level, as this was an exploratory analysis to demonstrate the methodology.

Finally, there are many other user impacts such as noise, safety, maintenance, repair and, tire wear-tear which are a function of not only IRI but also other factors such as vehicle type and localized distresses like potholes. However, in this study, only energy consumption was used as an indicator to show the impact of road condition on the users as an example impact^62,63^.

## Results

### Analysis of road condition in EJ and non-EJ communities

**Figure 4:**
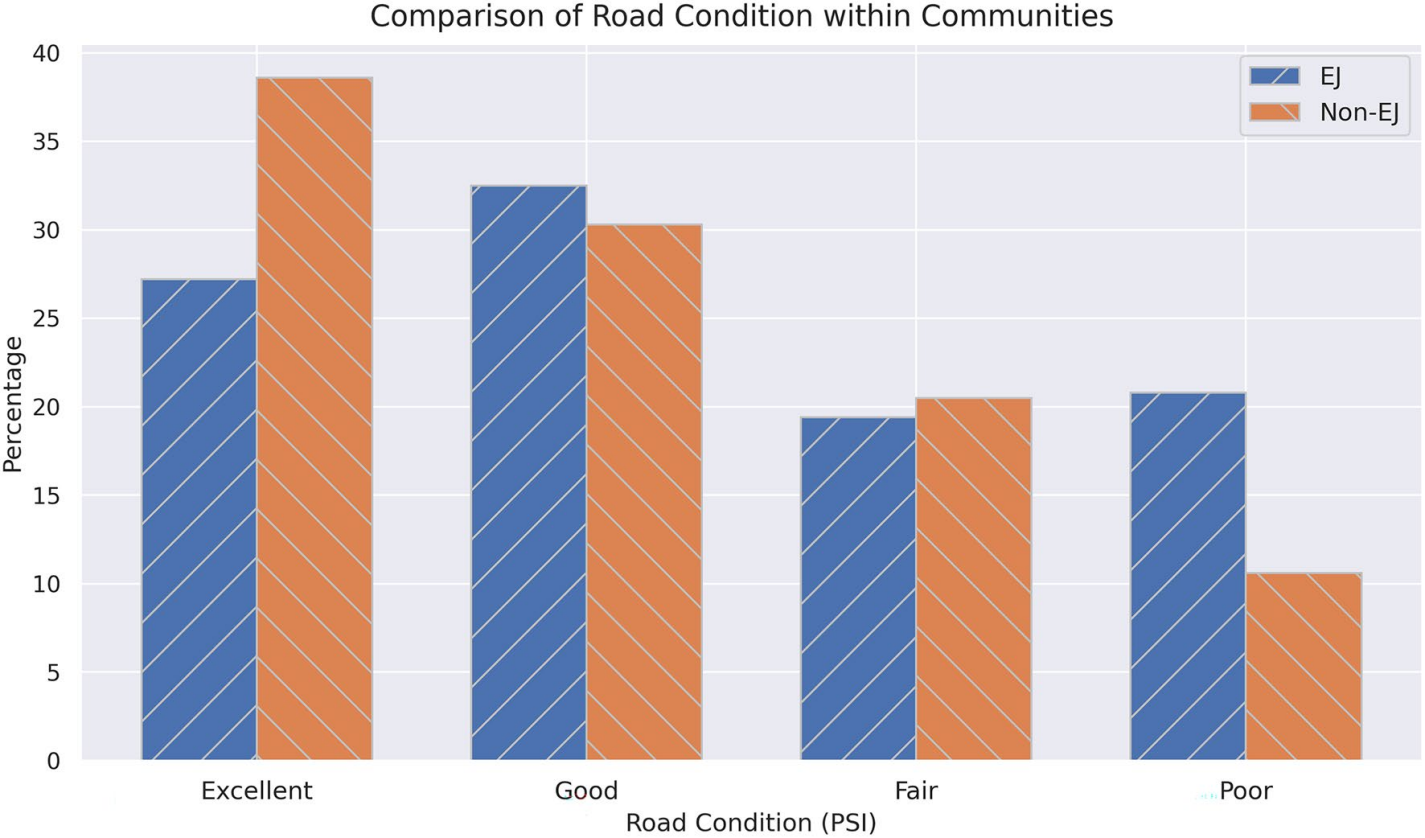
Road condition in EJ and non-EJ communities.

To achieve the first goal of this study, the road conditions that are within and outside EJ communities were compared. Figure 4 shows that non-EJ communities are much more likely to live near excellent roads compared to EJ communities (37% compared to 27%). Similarly, EJ communities are twice as likely to live near poor condition roads (21% compared to 10%). This shows that while the means and the medians of distributions are similar, there are disparities near the tails where road condition is either excellent or poor.

Given the established relationships between road emissions and increased health risks for communities that live near roadways^64,65^, this finding is extremely important and should be used to quantify the disproportional impacts of road condition on residents given that damaged road emissions are higher^66,67^. This is in addition to the findings showing that disadvantaged communities are more likely to be near interstates and other major roadways, creating overall unhealthy conditions for residents, not only in terms of emissions, but also more noise, less green space, and overall lower quality of life^31^. In the future, road condition disparity will be used to quantify negative health impacts due to pavement condition, which was out of the scope of this study.

### Analysis of recurrent home-work trips for EJ and non-EJ commuters

**Figure 5:**
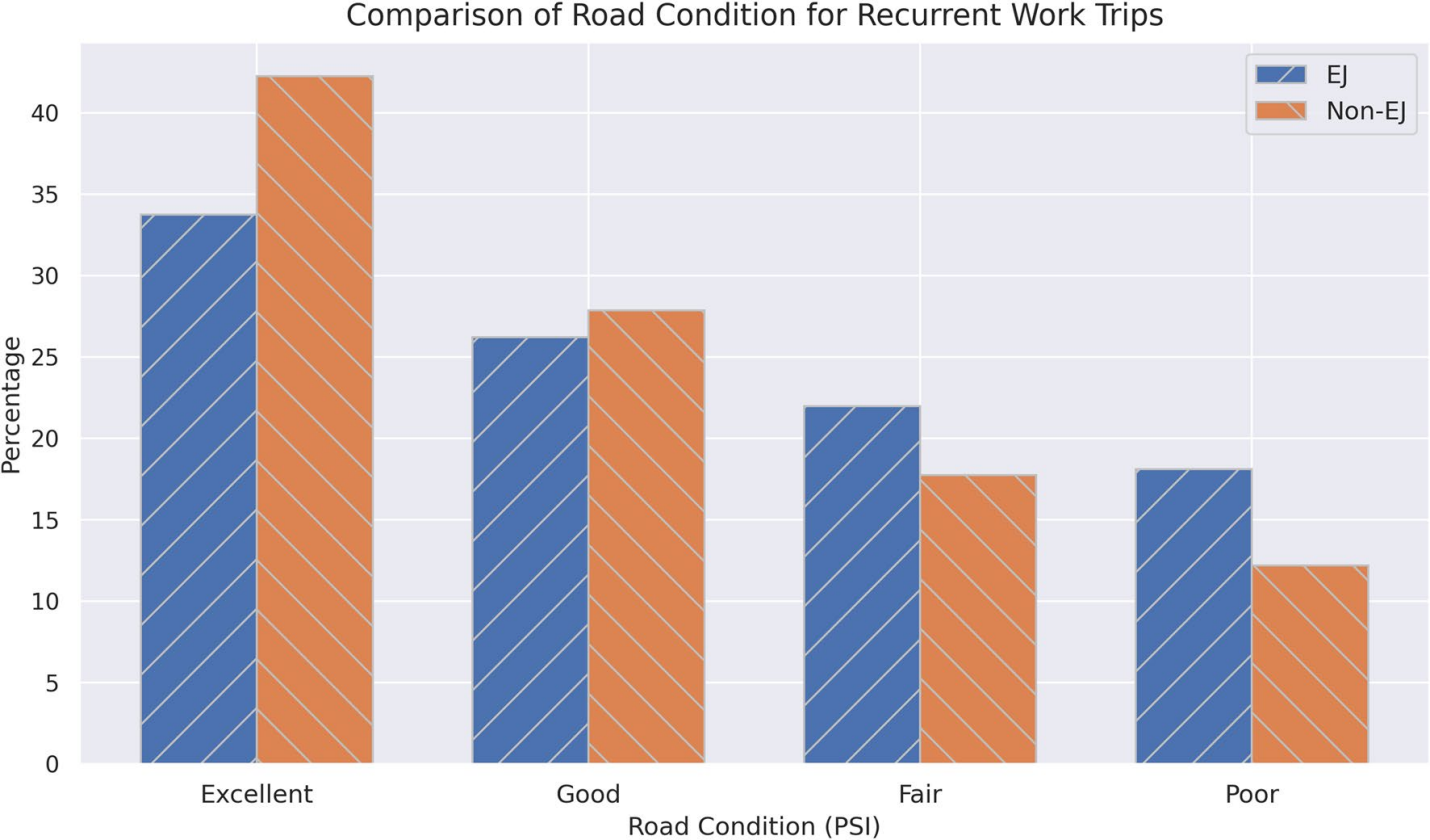
Road condition for EJ and non-EJ commuters’ trips.

While it is crucial to look at the condition of roads within a community, it is also important to realize that roads are an essential part of residents mobility. As the second goal of this study is to quantify the impact of road condition on recurrent trips, this study focused on daily home-work trips. Because these trips are essential and frequent, the impact of road condition on these trips are more important compared to less frequent trips such as trips to health care facilities or grocery stores. The condition of these roads not only affect the quality of life, but also the excessive fuel consumption. For EJ and non-EJ communities, Fig. 5 shows the average road conditions for these recurrent trips.

Similar to road condition within communities, non-EJ commuters are more likely to use excellent roads during their trips (42% compared to 34%), while those who reside in EJ communities are more likely to travel on poor roads (18% compared to 12%).

**Figure 6:**
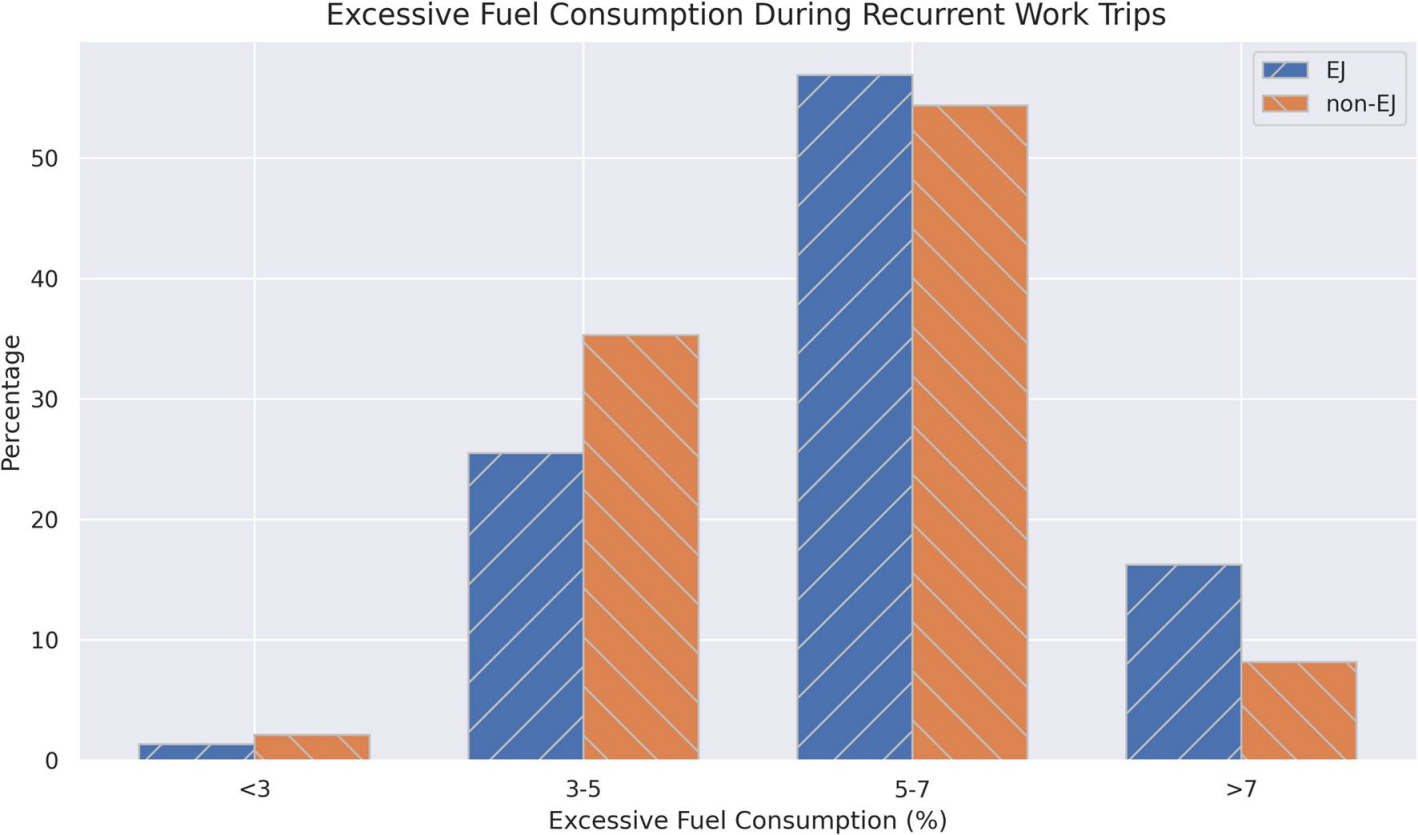
Excessive fuel consumption for EJ and Non-EJ commutes.

To quantify the impact of road condition on the trips, the IRI values were also computed for each trip segment. Becase EJ communities travel on rougher roads, their fuel consumption is also higher for these recurrent trips. Figure 6 demonstrates the excessive fuel consumption, calculated from Eq. (3).

The majority of Massachusetts commuters use over 3% excessive fuel/energy on their commutes to work which shows that for all communities, the road condition increases the road user costs. However, there are again disproportionate impacts on the EJ commuters. Many non-EJ commuters use less than 5% fuel compared to EJ commuters (39% compared to 27%) and double the EJ commuters use over 7% excessive fuel consumption compared to non-EJ commuters (16% compared to 8%). These results clearly indicate that the EJ residents are not only residing near poor condition roads, they also face disproportionate impacts during their essential and frequent trips.

## Discussion of future work

This study outlines a methodology for calculating the disproportionate impact of road condition for disadvantaged communities who live near and travel on these roads. Metrics such as Gini coefficient or the Theil index could be used to quantify such inequalities^39^ and the impact of different maintenance policies on inequality measures. Gini coeeficient measures the inequality among the values of a distribution, such as levels of fuel consumption or health impacts. A Gini coefficient of 0 reflects perfect equality, while a Gini coefficient of 1 reflects maximum inequality. Theil index is a measure of overall inequality, with high values indicating higher inequality^68^. There are other measures such as Hoover index or Atkinson’s index that can be tailored to measure inequality due to pavement condition. Alternatively, an agency may want to select a resolution and a threshold and calculate the disparity exceeding said threshold.

For incorporating the impact of road condition for nearby residents, the disproportionate health impacts could be calculated after considering other important inputs such as traffic volume and proximity to the roads. This can be done at a higher resolution, such as for a county, city network or a single section. In LCCA, while user costs (including emissions, safety, delay, etc.) are sometimes calculated, usually they are aggregated for all users. Using some inequality measures, the variation of these costs could be a part of LCCA. For LCA, the use stage could include an inequality measure for a given alternative in addition to the total emissions.

Although LCCA and LCA are powerful tools for quantifying economic and environmental impacts respectively, they fall short when the impacts cannot be clearly measured in monetary terms or emissions. In these cases, it is even more important to include additional social components in sustainability analyses that measure the overall social inequality of these impacts. Finally, the definition of disadvantaged communities may change from project to project and location to location depending on the type of analysis. The responsible agency must determine the definitions of disadvantages communities for a given analysis and state the criteria clearly.

## Data Availability

The source data used in this study can be found in^44,57,58^.

## Code availability

The code used to analyze the datasets can be found on github.com/egemenokte/PavementSocialImpact.
